# A Small RNA, UdsC, Interacts with the R*poHII* mRNA and Affects the Motility and Stress Resistance of *Rhodobacter sphaeroides*

**DOI:** 10.3390/ijms232415486

**Published:** 2022-12-07

**Authors:** Daniel-Timon Spanka, Julian Grützner, Andreas Jäger, Gabriele Klug

**Affiliations:** Institute of Microbiology and Molecular Biology, Justus Liebig University Giessen, Heinrich-Buff-Ring 26-32, 35392 Giessen, Germany

**Keywords:** sRNA UdsC, RpoHII sigma factor, bacterial motility, oxidative stress response, *Rhodobacter sphaeroides*

## Abstract

sRNAs have an important role in the regulation of bacterial gene expression. The sRNA, UdsC, of *Rhodobacter sphaeroides* is derived from the 3′ UTR of the RSP_7527 mRNA, which encodes a hypothetical protein. Here, we showed the effect of UdsC on the resistance of *Rhodobacter sphaeroides* to hydrogen peroxide and on its motility. In vitro binding assays supported the direct interaction of UdsC with the 5′ UTR of the *rpoHII* mRNA. RpoHII is an alternative sigma factor with an important role in stress responses in *R. sphaeroides*, including its response to hydrogen peroxide. We also demonstrated that RpoHII controls the expression of the *torF* gene, which encodes an important regulator of motility genes. This strongly suggested that the observed effect of UdsC on TorF expression is indirect and mediated by RpoHII.

## 1. Introduction

In most habitats, bacteria are exposed to frequent changes in their environment. *Rhodobacter sphaeroides* (now *Cereibacter sphaeroides* [1]) is a facultative phototrophic bacterium, which is mostly found in fresh and brackish water. It is able to perform aerobic or anaerobic respiration, fermentation or anoxygenic photosynthesis [2,3]. The formation of photosynthetic complexes is controlled by oxygen and light conditions to avoid the risk of photooxidative stress. This adaptation is accompanied by changes in the transcriptome, and the mechanisms of transcriptional regulation have been intensively investigated over the last decades. An important role in the regulation of photosynthesis gene expression in response to light and oxygen was revealed for the two-component system PrrB/PrrA [4], the repressor/antirepressor system PpsR/AppA [5,6,7] and the FnrL protein [8]. In addition, small non-coding RNAs such as PcrZ, PcrX and asPcrL affect the expression of photosynthesis genes [9,10,11]. Furthermore, regulators of the photo-oxidative stress response have been identified, namely the alternative sigma factors RpoE, RpoHI and RpoHII [12,13,14,15] as well as the sRNAs SorY, SorX and CcsR1-4 [16,17,18]. The number of known sRNAs with an important role in the regulation of physiological processes in bacteria is steadily increasing, and different mechanisms have been demonstrated for regulation through sRNAs (reviewed in, e.g., [19,20,21,22]).

Many sRNAs are derived from the 5′or 3′ UTRs of mRNAs either by partial termination, ribonucleolytic cleavage or the internal processing of the mRNA, or they can be transcribed by an own promoter (in the case of 3′ UTRs) (reviewed in, e.g., [20,21]). We recently identified eight so-far-unknown UTR-derived sRNAs in *R. sphaeroides* (UdsA-UdsH) and demonstrated that the maturation of sRNAs is modulated during adaptation to different growth conditions [23]. Consequently, sRNA maturation contributes to the regulation of gene expression.

The 67 nt sRNA UdsC is derived from the 3′ UTR of the RSP_7527 (RSP_RS22195) mRNA, which encodes a hypothetical protein. The ribonucleases RNase III, RNase E, PNPase and the RNA chaperone Hfq are involved in the maturation of UdsC. UdsC levels increase in the presence of hydrogen peroxide or singlet oxygen, decrease under heat stress or iron depletion and depend on RpoHII [23]. In order to learn more about the physiological role of UdsC, we compared the transcriptomes of a strain lacking UdsC and a strain overexpressing UdsC to that of the isogenic wild type. The RNAseq results revealed the effect of UdsC on the expression of genes related to motility, and physiological experiments confirmed the role of UdsC in motility. The overexpression of UdsC also affected resistance to oxidative stress.

## 2. Results

### 2.1. Effect of Deletion or Overexpression of UdsC on the Transcriptome of R. sphaeroides

**Figure 1:**
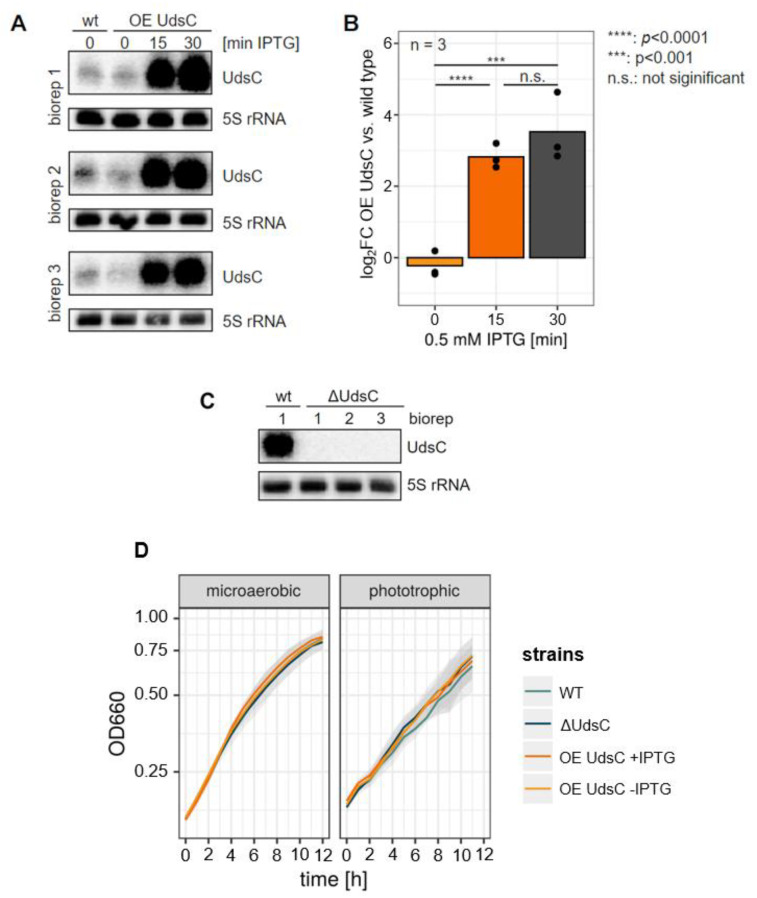
(**A**) Northern blots with total RNA (biological triplicates) from wild type (WT) or the UdsC-overexpression strain (OE UdsC) hybridized to an UdsC-specific probe (upper panels) or to 5S rRNA as loading control (lower panels). (**B**) The signals for UdsC were normalized to 5S rRNA and plotted for the different time points after IPTG addition. The bars represent the mean of the single measurements (dots), and the significance of the differences is indicated. (**C**) Northern blot from total RNA of the wild type (WT) and the strain deleted for UdsC (ΔUdsC) (biological triplicates) hybridized against UdsC (**upper** panel) or 5S rRNA (**lower** panel). (**D**) Growth curves of WT, UdsC knockout (ΔUdsC) and the overexpression strain (with or without IPTG added) under microaerobic and phototrophic conditions. The lines represent the mean of biological triplicates, and the standard deviations are indicated (shade).

To analyze the function of UdsC, we constructed a strain (ΔUdsC) that lacked the first 36 nucleotides of the DNA sequence for the sRNA but retained the coding region for RSP_7527 and the terminator (nucleotides 39 - 67) for the RSP_7527-UdsC locus. In addition, the *udsC* gene was cloned into a plasmid (pCV2_*udsC*) that allowed IPTG-induced overexpression in *R. sphaeroides* (strain OE UdsC). Figure 1A shows the levels of UdsC in the wild type and in the strain OE UdsC on a Northern blot. The RNA levels were normalized to 5S RNA, and the quantification in Figure 1B shows about a 12-fold overexpression of UdsC 30 min after IPTG addition. The Northern blot in Figure 1C confirmed the lack of UdsC in the deletion strain. Figure 1D demonstrates the very similar growth of all the strains under microaerobic conditions, which were applied for RNA isolation and the subsequent RNAseq analysis. As shown in Figure S1, the growth behavior was also similar in the stationary phase and in the outgrowth after different periods in the stationary phase in microaerobic conditions. The RNAseq was performed in triplicates, and for each triplicate, three independent cultures were collected.

**Figure 2:**
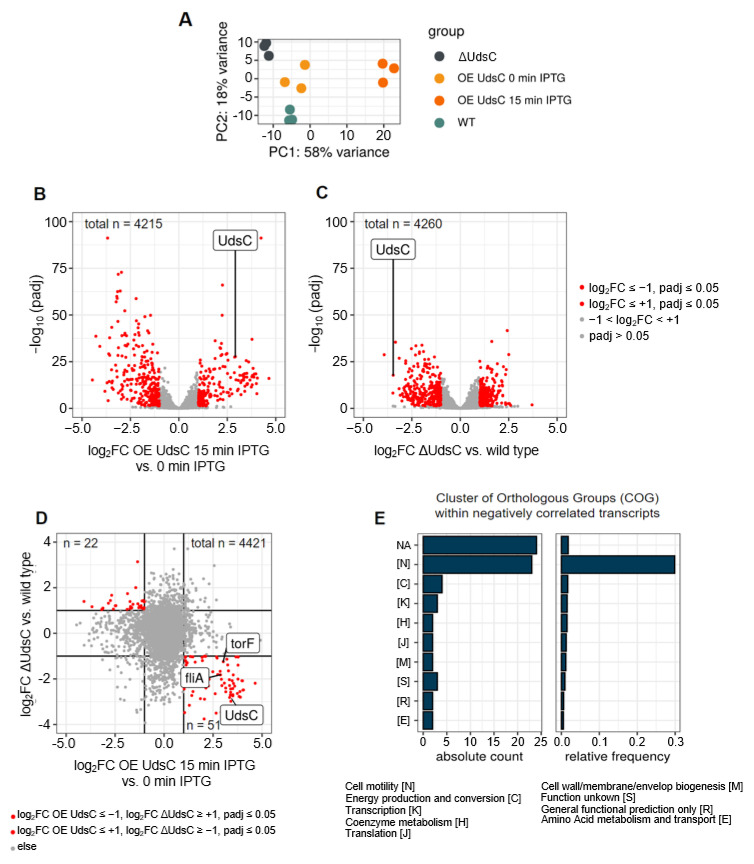
(**A**) PCA plot from the RNAseq data (triplicates) of the indicated strains. (**B**) Volcano plot comparing expression (RNAseq data) of the UdsC-overexpressing strain before and 15 min after addition of IPTG. Genes with significant changes in abundance are colored in red (log_2_fold change ≤ −1 or ≥+1, adjusted *p*-value ≤ 0.05, base mean ≥ 50) and in grey (−1 ≤ log_2_-fold change ≥ +1 and *p*-value > 0.05). Altogether, the transcripts of 4215 genes were observed to differ in a statistically significant manner and exhibited a base mean above the threshold. (**C**) Volcano plot comparing expression (RNAseq data) of the UdsC-deletion strain (ΔUdsC) and the wild type. Genes with significant changes in abundance are colored in red (log_2_fold change ≤ −1 or ≥+1, adjusted *p*-value ≤ 0.05, base mean ≥ 50) and in grey (−1 ≤ log_2_-fold change ≥ +1 and *p*-value > 0.05). Altogether, the transcripts of 4260 genes were observed to differ in a statistically significant manner and exhibited a base mean above the threshold. (**D**) Scatter plot comparing the expression change in the UdsC-overexpressing strain before and 15 min after addition of IPTG with the expression change in the UdsC mutant versus wild type. Genes marked in red are listed in Table S4. (**E**) All genes that are marked in red in Figure 2D (n = 73) were subjected to a COG analysis. The number of those genes in each cluster is shown in the left panel, and the right panel gives the percentage of genes in an affected cluster. Genes marked in red are listed in Table S4.

The PCA plot (Figure 2A) showed good reproducibility among all the triplicates and demonstrated that the transcriptome of OE UdsC after 15 min of IPTG addition clearly varied from the other strains and from the transcriptome of OE UdsC before the addition of IPTG.

The volcano plots in Figure 2B,C visualize the changes in the transcriptomes of the OE UdsC 15 min after the addition of IPTG and the changes in the wild type versus ΔUdsC. As was already suggested by the PCA plot, the transcriptome of OE UdsC 15 min after IPTG addition showed stronger differences to that of the control (OE UdsC without IPTG addition) than the transcriptome of ΔUdsC compared to the wild type.

Differential expression between the UdsC-deletion strain and the wild type and the over-expression strain grown with or without IPTG is compared in a scatter plot in Figure 2D and Table S4. Genes with a significantly different and opposite change in expression in the over-expression and in the deletion strain are marked in red (Figure 2D and Table S4). With all these genes, a COG analysis was performed (Figure 2E). The left panel shows the number of genes with a changed expression in each cluster, while the right panel gives the percentage of genes in affected a cluster. This analysis clearly revealed that genes for cell motility were especially affected by an altered level of UdsC (30% of the genes in this category were affected). Table 1 lists the 50 genes with the highest positive fold change in expression between the overexpression strain after 15 min of IPTG and with no exposure to IPTG, and the 50 genes with the highest negative fold change are listed in Table S3. Alongside this, the changes between ΔUdsC and the wild type are listed. For most of the genes with a higher expression in the overexpression strain exposed to IPTG, the change in gene expression in the mutant/wild type showed the opposite effect. This was to be expected if UdsC affected the expression of those genes. Of the 50 genes with highest positive fold change upon the overexpression of UdsC, 33 genes were related to motility based on their annotation. With exception of *motA* and *motB*, the motility-related genes were clustered on the chromosome of *R. sphaeroides* and organized in several operons (screen shot, Figure S2). RSP_0033, RSP_0035, RSP_0038, RSP_0067, RSP_0072, RSP_6086-RSP_6090 and RSP_6091 are hypothetical proteins. Their co-localization with the motility genes suggested that they also had a function in motility. If this assumption was true, 44 of the 50 genes with a higher expression upon overexpression of UdsC would have been related to motility. These genes showed a decreased expression in ΔUdsC compared to the wild type. However, some motility genes showed similar or even lower expression levels in OE UdsC after 15 min of exposure to IPTG compared to that with no exposure to IPTG (cluster D in Figure S2D). For example, the *fhlB* gene is part of an operon, together with RSP_1319, *fhlA1*, *fliR1* and RSP_6155, that is located in another region of the chromosome and shows an expression pattern different from the other motility genes.

Among the 50 genes with lowest fold change upon overexpression of UdsC were 13 genes related to photosynthesis. However, an influence of UdsC expression on phototrophic growth was not observed (Figure 1D).

### 2.2. Effect of UdsC on Motility and Resistance to Hydrogen Peroxide

**Figure 3:**
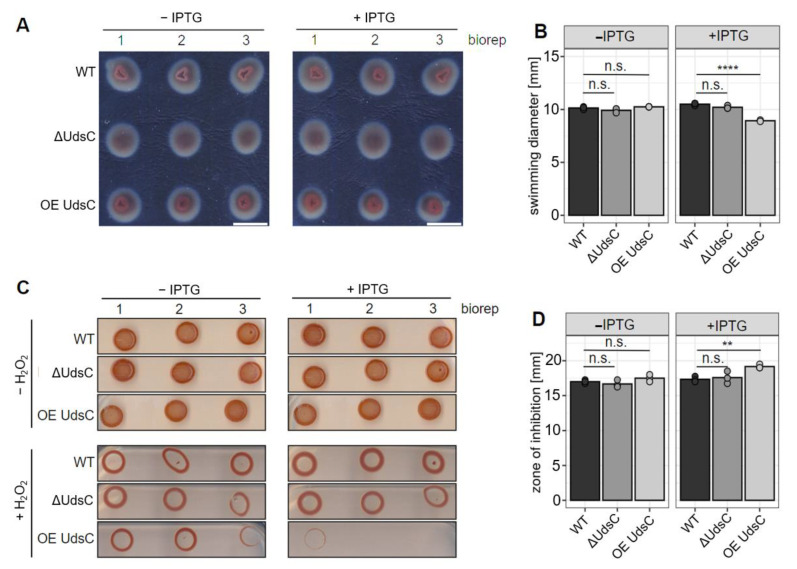
(**A**) Swimming assays of the indicated strains in absence or presence of the inducer, IPTG (biological triplicates). (**B**) Diameters of the assay shown in (**A**). The bars give the average diameter of the triplicates (points). n.s.: not significant, ****: padj ≤ 0.0001. (**C**) Spot assay of the indicated strains in absence or presence of the inducer, IPTG (0.5 mM IPTG; biological triplicates), and in presence or absence of hydrogen peroxide (1 mM). (**D**) Quantification of zone of inhibition assays (not shown) in presence of hydrogen peroxide (1 mM). The bars give the average diameter of the triplicates (points). n.s.: not significant, **: padj ≤ 0.01.

Since the RNAseq results revealed a strong effect of UdsC on motility genes, we performed swim assays with the different strains (Figure 3A). The quantification of triplicates from the swimming assays revealed a slight, but not significant, motility-stimulating effect of IPTG (Figure 3B). The ΔUdsC strain showed a similar motility to the wild type, while the motility of the overexpression stain was moderately, but significantly, decreased in the presence of IPTG (Figure 3B). This demonstrated, that UdsC indeed affected motility.

A previous study demonstrated that the levels of UdsC are strongly increased by hydrogen peroxide treatment [23]. Therefore, we also tested the effect of UdsC on resistance to hydrogen peroxide. The spot assay in Figure 3C clearly demonstrates a lower resistance of *R. sphaeroides* to hydrogen peroxide when UdsC was overexpressed. In addition, we performed zone of inhibition assays that confirmed this result (Figure 3D).

### 2.3. UdsC Targets the mRNA for the Alternative Sigma Factor RpoHII

How does UdsC act on motility and stress resistance? Most sRNAs affect gene expression by binding to mRNA targets and by either influencing their rate of translation, their rate of decay or both [24]. To get an idea about putative targets of UdsC, we applied the IntaRNA tool [25] in order to conduct search against the genome of *R. sphaeroides*.

*RpoHII* mRNA was suggested as a putative target and ranked at position seven in the IntaRNA search based on the IntaRNA energy value, whereas *TorF* was ranked at position nineteen [25]. RpoHII is known as main regulator in the oxidative stress response of *R. sphaeroides*, and TorF (FleT, RSP_0051) is known as regulator of class III flagellar genes together with RpoN2 and FleQ [26,27]. *TorF* was among the 50 most increased mRNAs in the UdsC-overexpression strain after 15 min of IPTG treatment (Table 1). The gene is part of the motility gene cluster shown in Figure S2. The *fleQ* mRNA increased by a log_2_fold of 1, and the *rpoN2* mRNA increased by a log_2_fold of 2.8 upon the overexpression of UdsC. The *RpoHII* mRNA showed threefold higher levels in the RNAseq in strain ΔUdsC compared to the wild type. Based on the IntaRNA prediction and the expression profiles, we further investigated the effect of UdsC on *TorF* and *RpoHII* expression.

**Figure 4:**
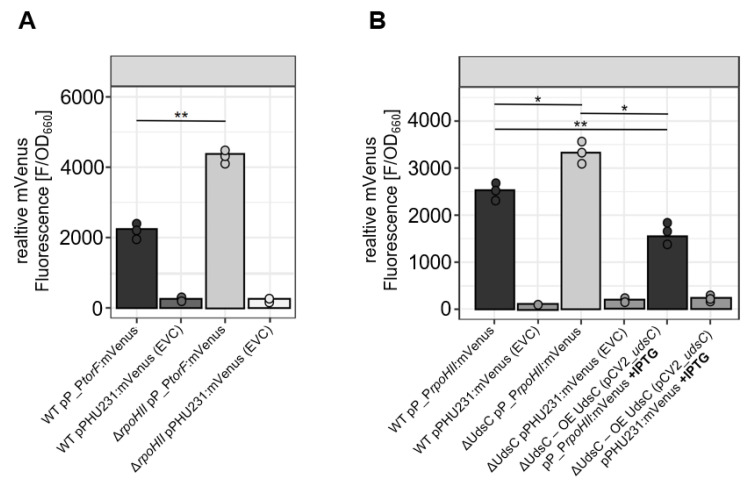
Effect of RpoHII on TorF expression and of UdsC on *RpoHII* and *TorF* expression (**A**) The promoter region of *TorF* was fused to mVenus on plasmid pP_p*torF*:mVenus, which was conjugated into wild-type *R. sphaeroides* (WT) or into the RpoHII-deletion strain (Δ*RpoHII*), respectively. The promoter activity (fluorescence) was measured in cultures grown under microaerobic conditions. The bars represent the mean of the single measurements (dots), and the significance of the differences is indicated (**: padj ≤ 0.01). (**B**) The promoter region of *rpoHII* was fused to mVenus on plasmid pP_p*rpoHII*:mVenus), which was conjugated into wild-type *R. sphaeroides* (WT), the UdsC-deletion strain (ΔUdsC) or the UdsC-deletion strain with an inducible UdsC complementation (ΔUdsC–OE UdsC). The cultures were incubated under microaerobic conditions, and the expression of UdsC was induced by 0.5 mM IPTG. The bars represent the mean of the single measurements (dots), and the significance of the differences is indicated (*: padj ≤ 0.05, **: padj ≤ 0.01).

The UdsC binding site, as predicted by IntaRNA, was localized in the 5′ UTR of *RpoHII*, starting only two nt downstream of its main 5′ end. The predicted binding site for *TorF* was located directly upstream of the transcriptional start, which would exclude a binding of UdsC to *TorF* mRNA (Figure S3). Further inspection of the sequence around the *TorF* transcriptional start site revealed a high similarity to the RpoHII recognition site [13], suggesting that the effect of UdsC on TorF may be indirect and mediated by RpoHII. To support this assumption, a *torF*:mVenus reporter construct was transferred into the wild type or into a strain lacking RpoHII. As shown in Figure 4A, a lack of RpoHII led to a twofold higher activity of the reporter, confirming an influence of RpoHII on *TorF* expression.

To further test the influence of UdsC on Tor*F* and *RpoHII* expression, we cloned the upstream region of the genes into reporter plasmids, pCV2, with the mVenus gene fused to the translational starts. There was no significant effect of the deletion or overexpression of UdsC on *TorF* expression (data not shown), whereas *rpoHII*-mVenus expression was slightly (about 1.2-fold) higher in the ΔUdsC strain than in the wild type. When UdsC was overexpressed, *rpoHII*-mVenus expression was only 50% of that in the ΔUdsC strain and 63% of that in the wild type. From these results, we excluded a direct effect of UdsC on *TorF* expression but considered that *RpoHII* expression was directly affected by UdsC, while *TorF* expression was affected by RpoHII.

**Figure 5:**
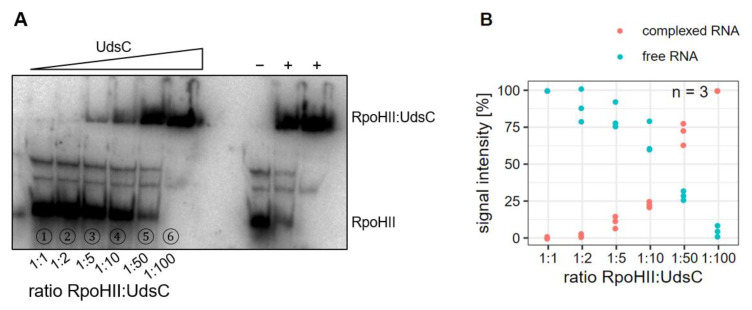
(**A**) Gel retardation assay of an *RpoHII* segment (93 nt) and UdsC (70 nt). ^32^P-labelled *RpoHII* in vitro transcripts [150 fmol] were incubated together with increasing concentrations of unlabeled UdsC in vitro transcript. Samples were loaded onto a 6% non-denaturing polyacrylamide gel containing 0.5× TBE. (**B**) Quantification of the free and complexed RNA from three independent gel retardation assays by phosphoimaging.

The predicted interaction between *RpoHII* mRNA and UdsC is shown in Figure S3. To further validate *RpoHII* mRNA as target of the sRNA UdsC, we applied electromobility shift assays to test for a direct interaction of the two RNAs. A 93 nt radio-labelled in vitro transcript containing the *RpoHII* promoter region and the predicted UdsC binding site was incubated with increasing amounts of a UdsC (70 nt) in vitro transcript. Figure 5 A shows that UdsC formed a complex with *RpoHII* mRNA, resulting in reduced migration in the gel. The intensities of the shifted and non-shifted bands of triplicates were quantified and plotted (Figure 5B). Our results demonstrated a direct interaction between UdsC and *RpoHII* mRNA.

## 3. Discussion

Numerous sRNAs have been identified in bacteria over the last years, and for some of them important roles in the regulation of gene expression and their effects on important physiological functions were demonstrated [24,28,29,30]. For the majority of sRNAs, their function is still unknown. For the 3′ UTR-derived sRNA, UdsC, from *R. sphaeroides,* an influence of certain stress factors on its abundance was demonstrated [23], suggesting that it may have a function in stress responses. To better understand the function of UdsC, we constructed strains lacking or overexpressing this sRNA and performed physiological tests and a comparative RNAseq analysis.

The RNAseq analysis revealed a strong effect of UdsC on the expression of motility genes, and an effect of UdsC on motility was confirmed. What is the molecular basis for the regulation of motility genes by UdsC? Many motility genes are clustered in the chromosome of *R. sphaeroides* (Figure S2) and TorF (FleT), the Fis family protein, FleQ, and the alternative sigma factor, RpoN2, are their main regulators [26,27]. TorF improves binding of FleQ to DNA [27]. The IntaRNA tool suggested a base pairing between UdsC and *TorF*. However, the predicted UdsC binding site was located upstream of the transcriptional start site of *TorF,* and the reporter assays showed no direct influence of UdsC on the *TorF* promoter. A good binding site of UdsC was also predicted for the *TpoHII* 5′ UTR, and the reporter assay confirmed that *RpoHII* promoter activity was influenced by UdsC. An interaction between the *RpoHII* 5′ UTR and UdsC was also supported by an electromobility assay. RpoHII is known as an important regulator in several stress responses of *R. sphaeroides*, e.g., oxidative stress, heat stress and stationary phase [12,13,14,31,32,33]. It activates a rather large regulon that partially overlaps with the RpoHI regulon [34]. RpoHI expression was not affected by UdsC deletion or overexpression (Table/RNAseq data). The promoter of *TorF* had a high similarity to RpoHII-dependent promoters, however the distance between the −10 and −35 regions was 2 nt shorter. Nevertheless, a RpoHII-dependent expression of *TorF* was confirmed by the reporter assay, but this effect may well be indirect.

Although the role of TorF in the regulation of motility genes is well established, a TorF regulon has not been defined [27]. Many motility genes were induced upon UdsC overexpression, whereas others were unaffected or even showed decreased expression. It is not known whether this was due to the different effects of TorF on motility genes or to the TorF-independent effects of UdsC. The promoter of RSP_7527*-udsC* is RpoHII-dependent [23], and increased levels of UdsC repress *RpoHII* expression (as shown in this study), suggesting a negative feedback loop.

Due to its established role in stress responses, the UdsC-dependent expression of *RpoHII* could well explain the role of UdsC in stress responses. However, genes of the RpoHII regulon respond differently to a lack of UdsC or its overexpression. For example, the small RpoHI/HII-dependent sRNAs SorY [18] and StsR [35] showed decreased levels upon overexpression of UdsC, while the RpoHII-dependent RSP_3164 and RSP_0150 [13] mRNAs showed increased levels (Table 1). Other RpoHII- or RpoHI/HII-dependent genes did not respond to an altered level of UdsC. Hence, the increased levels of *RpoHII* upon lack of UdsC did not lead to the expected increased expression level of all RpoHII-dependent promoters. This further indicated that so-far-unknown factors were involved in the UdsC-dependent signal transduction. Differences in promoter strength or additional protein regulators may be such factors.

## 4. Materials and Methods

### 4.1. Bacterial Strains and Growth Conditions

The bacterial strains and plasmids used in this study are listed in Supplementary Table S1. Microaerobic *Rhodobacter sphaeroides* cultures were incubated in Erlenmeyer flasks under microaerobic conditions. The 50 mL flasks were filled with 40 mL malate minimal medium and sealed with a cellulose plug. The cultures were shaken at 140 rpm to achieve a dissolved oxygen concentration of 25–30 μM [31]. The oxygen concentration was measured in the cultures by a Geisinger Electronics GMH3610 oxygen meter. Cultivation of liquid cultures was performed at 32 °C in the dark. When necessary, spectinomycin (10 µg/mL) or gentamicin (10 µg/mL) was added to the liquid growth media. Pre-cultures and experimental main cultures of the UdsC-overexpression strain were supplemented with 0.2 µM crystal violet (CV) [36]; the overexpression was induced during the exponential growth phase by adding Isopropyl β-d-1-thiogalactopyranoside (IPTG) to a final concentration of 0.5 mM. Phototrophic growth was carried out in completely filled airtight sealed Metplat bottles (screw cap). The cultures were exposed to white light with an intensity of 40 W/m^2^ at 32 °C. The strains used in this study were the wild-type *Rhodobacter sphaeroides* 2.4.1, a UdsC-overexpression (OE UdsC) strain harboring the pCV2_*udsC* and a *UdsC1-36*-deletion strain (see below).

### 4.2. Construction of the UdsC Deletion Strain

Bases 1 – 36 of *UdsC* were deleted in the wild-type *Rhodobacter sphaeroides* 2.4.1 strain using a scarless deletion strategy based on the plasmid pK18mobII. The approximately 600 bp long upstream (primers 7527_sRNA_up_EcoRI_for/7527_sRNA_up_XbaI_rev) and downstream (primers 7527_sRNA_dn_XbaI_for/7527_sRNA_dn_HindIII_rev) regions were amplified by PCR and cloned to pK18mobII using the restriction enzymes EcoRI/XbaI (upstream fragment) and XbaI/HindIII (downstream fragment). Plasmids were transformed to *E. coli* strain S17–1 and subsequently transferred to *Rhodobacter sphaeroides* 2.4.1 by diparental conjugation [37]. After conjugation, clones were selected on malate-minimal agar containing 25 µg/mL kanamycin. Next, cells from single colonies were transferred to (a) malate-minimal agar containing 5% (*w*/*v*) sucrose and to (b) malate-minimal agar containing 25 µg/mL kanamycin. Positive clones that did not grow in the presence of sucrose were selected and grown in malate-minimal liquid cultures under microaerobic conditions at 32 °C overnight. The cultures were subsequentially spread on 5% (*w*/*v*) sucrose malate-minimal agar and cultivated for three days. Single colonies were then transferred to (a) malate-minimal agar containing 25 µg/mL kanamycin and (b) 5% (*w*/*v*) sucrose malate-minimal agar. Positive clones were verified by PCR and DNA sequencing. Primer sequences are provided in Supplementary Table S2.

### 4.3. Construction of the UdsC-Overexpression Plasmid

The gene *UdsC* including its native rho-independent terminator structure was amplified with the primer pair 7527_sRNA_SacI_for/7527_sRNA_mRNA_KpnI_rev and then cloned to the pCV2 plasmid with the restriction enzymes SacI and KpnI. Plasmid pCV2 was based on the pBBR1 plasmid [38] that was modified by inserting the *lacI* gene from *E. coli* and a LacI-repressed (IPTG inducible) promoter. This combination of *lacI* and an inducible promoter has been previously used in the suicide vector pK18mobII that was inserted into the chromosome and maintained with antibiotics to control the expression of *mraZ* [35]. Plasmid pCV2 was a gift from Dr. Matthew McIntosh and is currently being included in a manuscript for publication. Briefly, the plasmid consisted of the vector pBBR1 plus three DNA fragments. The first fragment (*EcoRI*/*HindIII*) contained the Eil system consisting of the gene for a transcription repressor, *EilR*, and Peil, the promoter repressed by EilR. Repression by EilR was inactivated by the inducer crystal violet [36]. The second fragment (*HindIII*/*NdeI*) contained the *lacI* gene and P16S-O, the promoter repressed by LacI. Repression of P16S-O by LacI was inactivated by the inducer IPTG. The third fragment (*NdeI*/*SacI*) contained the gene for the transcription activator *sinR* and PsinI, a promoter that is strictly only activated by SinR [39]. The combination of these three fragments ensured tight control over transcription of the gene of interest, where the addition of crystal violet resulted in repression of the gene of interest, and the addition of IPTG activated strong expression (regardless of the presence of crystal violet). Also important was the fact that the transcription start of PsinI was so constructed with respect to a *SacI* restriction digest site so that the resulting RNA product of the gene of interest (cloned using *SacI*/*KpnI*) did not include any additional nucleotides (as is the case with promoters controlled by Lac operators). Thus, the expression of the gene *UdsC* was tightly controlled by the presence of crystal violet and IPTG. The final construct, pCV2_*udsC,* was transformed to *E. coli* S17–1 and conjugated to the wild-type *R. sphaeroides* 2.4.1 strain (OE UdsC) [37]. The inducible overexpression was verified via Northern blot analysis with a specific probe directed against UdsC and via total RNA sequencing. Primer sequences are provided in Supplementary Table S2.

### 4.4. RNA Isolation and Northern Blot Analysis

Total RNA isolation was performed using the hot phenol method [40]. Remaining DNA was subsequentially digested by a DNase treatment (#AM1907, Invitrogen^TM^, Vilnius, Lithuania 02241), which was conducted according to the manufacturer’s protocol. The electrophoretic separation and Northern blotting were performed as described earlier [41]. Oligonucleotides were end-labelled using the T4 polynucleotide Kinase (T4-PNK, Thermo Scientific^TM^, Vilnius, Lithuania 02241) and radioactive [γ^32^P]-ATP (Hartmann Analytics). A list of all the oligonucleotides used is provided in Supplementary Table S2. The QuantityOne 1-D Analysis Software (BioRad, version 4.6.6) was used for signal quantification.

### 4.5. In Vitro Transcription and EMSA

RNA was transcribed in vitro using T7 Polymerase (NEB) and PCR products as a template (primers listed in Table S2, which contained the T7 promoter region at the 5′ ends). Gel retardation assays were carried out with 150 fmol radio-labelled *RpoHII* transcript and various molar ratios of non-labelled *UdsC* transcripts in a final volume of 7.5 µL. RNAs were denatured separately for 1 min at 95 °C and renatured by cooling for 2 min on ice for 5 min at 32 °C. After these de- and renaturing steps, the radio-labelled and non-labelled RNAs were mixed, and 2 µL of 5× structure buffer (25 mM MgCl_2_ and 300 mM KCl) was added to a final volume of 10 µL. For complex formation of the RNAs, the samples were incubated together for 30 min at 32 °C. Afterwards, the reactions were mixed with 3 µL of loading dye (50% glycerol, 0.5× TBE, 0.2% bromophenol blue) and loaded onto a 6% non-denaturing polyacrylamide gel containing 0.5× TBE. Gels were pre-run at 100 V for 60 min at room temperature before loading. Electrophoresis was performed at room temperature by applying 200 V for 4 h. Gels were dried and exposed on phosphoimaging screens.

### 4.6. Spot Assay

To test the growth of the described strains in presence of H_2_O_2_, a spot assay was performed. Briefly, a 5 µL volume of exponentially growing cultures was placed on malate-minimal agar containing 1 mM H_2_O_2_ and/or 0.5 mM IPTG. Crystal violet was added to all plates to a final concentration of 0.2 µM. The petri dishes were incubated at 32 °C in the dark for two days.

### 4.7. Motility Assay

Exponentially growing cultures (5 µL each) were spotted on 0.15% (*w*/*v*) soft agar. The soft agar was supplemented with crystal violet (CV) and/or IPTG as described above. The petri dishes were incubated in a sealed plastic box containing wet tissues for 40 h at 32 °C in the dark.

### 4.8. Measurement of Sensitivity against H_2_O_2_

Sensitivity of the described strains against H_2_O_2_ was tested with a zone of inhibition assay as described by [42]. A mixture of 0.2 mL exponentially growing culture with 5 mL warm-top agar (0.8% *w*/*v*) was poured on a base layer of 15 mL malate-minimal medium in petri dishes. After cooling, a filter disc with 5 µL of a 1 M H_2_O_2_ solution was placed in the middle of the agar. Petri dishes were incubated for 48 h at 32 °C, and the diameters measured.

### 4.9. Library Preparation and RNA Sequencing

Three different precultures were inoculated with cell material from three individual colonies and grown under microaerobic conditions. These cultures were used to inoculate the experimental main cultures. Samples were taken during the exponential growth phase, and total RNA was isolated using the hot phenol method. Remaining DNA was digested via DNase treatment according to the manufacturer’s instructions (Invitrogen #AM1907). RNA quality was checked using a 2100 Bioanalyzer with an RNA 6000 Nano kit (Agilent Technologies). cDNA libraries suitable for sequencing were prepared from 100 ng of total RNA treated with T4 PNK for phosphorylation/dephosphorylation and RppH for decapping followed by NEBNext^®^ Multiplex Small RNA Library Prep (New England Biolabs) without fragmentation and rRNA depletion. The number of PCR cycles was determined to be 15 by qPCR, and the elongation time was set to 90 sec. Libraries were quantified by a QubitTM dsDNA HS Assay Kit 3.0 Fluometer (ThermoFisher), and quality was checked using a 2100 Bioanalyzer with a High-Sensitivity DNA kit (Agilent Technologies) before pooling. Sequencing of pooled libraries, spiked with 10% PhiX control library, was performed in single-end mode with a 75 nt read length on the NextSeq 500 platform (Illumina) with 2 High Output Kits v2.5. Demultiplexed FASTQ files were generated with bcl2fastq2 v2.20.0.422 (Illumina). The sequencing data are available at NCBI Gene Expression Omnibus (http://www.ncbi.nlm.nih.gov/geo, accessed on 25 October 2022) under the accession number GSE200990 and GSE216116

## 5. Bioinformatical Analysis

The adaptor removal and quality trimming of the raw reads was performed using Trimgalore (version 0.6.3) based on Cutadapt (version 2.4) with the parameters *--length 15 -j 8*. The trimmed reads were mapped on the *Rhodobacter sphaeroides* 2.4.1 genome (assembly GCF_000012905.2: NC_007493.2, NC_007494.2,NC_009007.1, NC_007488.2, NC_007489.1, NC_007490.2 and NC_009008.1) using the READemption pipeline (version 0.4.3; [43]) with the mapper segemehl (version 0.2.0; [44]). The differential gene expression analysis was carried out with the R package DESeq2 (version 1.26.0; [45]). READemption’s subcommand *coverage* was used to generate the nucleotide-wise coverage files.

## Figures and Tables

**Table 1:** Log_2_fold changes (calculated by DEseq2 analysis) in read counts determined by RNAseq within a UdsC-overexpression strain (OE UdsC) after 15 min of IPTG treatment in comparison to the UdsC-overexpression strain without (0 min) IPTG treatment and the UdsC-knockout strain (ΔUdsC) in comparison to the wild type (WT). The table shows the top 50 upregulated genes for the UdsC overexpression strain with exposure to IPTG (15 min) versus that without exposure to IPTG (0 min) in the fourth column. *Torf* and *UdsC* are marked in red.

Locus	Gene	Function	Log2FC 15 min IPTG vs. 0 min IPTG	Log2FC ΔUdsC vs. WT
RSP_2795	*RSP_2795*	Putative regulatory protein of multicomponent monooxygenase	4.92	−0.30
RSP_0083	*flgB*	Flagellar proximal rod protein, FlgB	4.65	−2.19
RSP_6228	*RSP_6228*	Hypothetical protein	4.24	−0.54
RSP_0034	*flhA*	Flagellar biosynthesis protein, FlhA	4.05	−0.80
RSP_0036	*flgA*	Flagellar basal-body P-ring formation protein, FlgA	4.02	−2.49
RSP_0063	*fliP*	Flagellar transport protein, FliP	4.00	−2.08
RSP_0061	*fliN*	Flagellar motor switch protein, FliN	3.97	−2.08
RSP_0067	*RSP_0067*	Hypothetical protein	3.89	−2.80
RSP_0035	*RSP_0035*	Hypothetical protein	3.87	−2.56
RSP_0082	*flgC*	Flagellar basal-body rod protein, FlgC	3.83	−2.77
RSP_0055	*fliH*	Flagellar protein, FliH	3.79	−2.08
RSP_0056	*fliI*	FliI, flagellum-specific ATPase	3.78	−1.04
RSP_0233	*motA*	Flagellar motor protein, MotA	3.77	−1.40
RSP_0058	*fliK*	FliK, flagellar hook-length control protein	3.77	−2.01
RSP_0062	*fliO*	Flagellar protein, FliO	3.74	−2.64
RSP_0057	*fliJ*	Flagellar protein, FliJ	3.67	−1.04
RSP_0066	*flhB*	Flagellar protein, FlhB	3.60	−0.21
RSP_0060	*fliM*	Flagellar switch protein, FliM	3.54	−2.20
RSP_6093	*flgJ*	Peptidoglycan hydrolase, FlgJ	3.53	−1.83
RSP_0231	*motB*	Flagellar protein, MotB	3.51	−2.70
RSP_0078	*flgG*	Flagellar distal rod protein	3.51	−2.74
RSP_0072	*RSP_0072*	Possible invasion protein	3.50	−2.42
RSP_0077	*flgH*	Flagellar L-ring protein	3.48	−2.08
RSP_0080	*flgE*	Flagellar hook protein, FlgE	3.41	−2.98
RSP_0033	*RSP_0033*	Hypothetical protein	3.40	−2.59
RSP_0074	*flgK1*	FlgK, flagellar hook-associated protein 1	3.40	−2.44
RSP_0430	*cobD*	Cobalamin biosynthesis protein, CobD	3.39	0.28
RSP_0064	*fliQ*	Flagellar protein, FliQ	3.38	−2.71
RSP_0081	*flgD*	Flagellar scaffolding protein, FlgD	3.35	−2.44
RSP_3794	*RSP_3794*	Hypothetical protein	3.34	0.41
RSP_6092	*RSP_6092*	Hypothetical protein	3.34	−2.29
RSP_6086	*RSP_6086*	Hypothetical protein	3.33	−2.04
RSP_0065	*fliR*	Flagellar protein, FliR	3.28	−1.65
RSP_0054	*fliG*	Probable flagellar motor switch protein, FliG	3.24	−1.39
RSP_0053	*fliF1*	Flagellar M-ring protein, FliF	3.2	−1.13
RSP_0052	*fliE*	Flagellar protein, FliE	3.19	−0.83
RSP_0076	*flgI*	Flagellar P-ring protein	3.15	−2.57
RSP_0059	*fliL*	Flagellar biosynthesis protein	3.14	−2.40
RSP_0073	*flgL*	Flagellar hook-associated	3.07	−2.19
RSP_0038	*RSP_0038*	Hypothetical protein	3.06	−0.90
**RSP_0051**	* **torF** *	**TorF protein**	**3.0**	−1.25
RSP_1641	*RSP_1641*	Hypothetical protein	2.96	−0.16
RSP_0079	*flgF*	Flagellar proximal-rod protein, FlgF	2.94	−0.26
RSP_3628	*RSP_3628*	Hypothetical protein	2.94	−2.02
RSP_3020	*RSP_3020*	Hypothetical protein	2.92	0.11
**NA**	* **udsC** *	**UdsC sRNA**	**2.91**	−1.82
RSP_0032	*fliA*	Sigma factor, FliA (Sigma-28)	2.87	−1.67
RSP_0084	*RSP_0084*	Hypothetical protein	2.86	−1.81
RSP_3387	*RSP_3387*	TRAP-T family transporter	2.73	−1.09
RSP_3017	*RSP_3017*	Nitrilotriacetate monooxygenase	2.7	−0.30

## Data Availability

The RNAseq data are available in the NCBI gene expression omnibus repository (GEO accession number GSE200990 and GSE216116).

## Supplementary Materials

The following supporting information can be downloaded at: https://www.mdpi.com/article/10.3390/ijms232415486/s1, Table S1: Bacterial strains and plasmids used in this study; Table S2: Oligonucleotide sequences for cloning, northern blot and in vitro transcription; Table S3: Log_2_fold changes (calculated by DEseq2 analysis) in read counts determined by RNAseq within a UdsC overexpression strain (OE UdsC) after 15 min of IPTG treatment in comparison to the UdsC overexpression without (0 min) IPTG treatment and the UdsC knockout strain (ΔUdsC) in comparison to the wild type (WT). The Table shows the top 50 downregulated genes for the UdsC overexpression strain with IPTG (15 min) versus without IPTG (0 min). Table S4: Log_2_fold changes (calculated by DEseq2 analysis) in read counts determined by RNAseq within a UdsC overexpression strain (OE UdsC) after 15 min of IPTG treatment in comparison to the UdsC overexpression without (0 min) IPTG treatment and the UdsC knockout strain (ΔUdsC) in comparison to the wild type (WT). The Table shows the 22 and 51 genes from Figure 2D, wich are colored in red. Table S5: Log_2_fold changes (calculated by DEseq2 analysis) in read counts determined by RNAseq within a UdsC overexpression strain (OE UdsC) after 15 min of IPTG treatment in comparison to the UdsC overexpression without (0 min) IPTG treatment and the UdsC knockout (ΔUdsC) in comparison to the wild type (WT). The Table shows 27 genes of the motility gene cluster that are not or slightly regulated. Figure S1: Growth curves of WT, UdsC knockout strain (ΔUdsC) and the UdsC overexpression strain (OE UdsC strain (with or without IPTG added) under microaerobic conditions. The cultures were incubated for 72 h. 24, 48 and 72 h after inoculation, part of the cultures was diluted into fresh medium and outgrowth was monitored, while the remaining culture was further incubated for monitoring the OD. The lines represent the mean of biological triplicates and the standard deviations are indicated (shade). Figure S2: Effect of UdsC on expression of the motility genes from RNAseq visualized by the Integrated Genome Browser. Wild type, UdsC deletion strain (ΔUdsC) and UdsC overexpression (OE UdsC) with and without IPTG (0 min IPTG or 15 min IPTG) were cultivated under microaerobic conditions and total RNA was isolated for RNA sequencing as described in materials and methods. The Log_2_fold change (Log_2_FC) of UdsC overexpression strain (OE UdsC) with IPTG (15 min) versus without IPTG (0 min) is idicated. Genes for important regulators of the flagella genes are marked in red. Figure S3: (**A**) Predicted base pairing between UdsC and *rpoHII* mRNA transcript by IntaRNA webtool [8]. The transcription start site (TSS) is indicated by an arrow and the start codon AUG is shown in red. (**B**) The predicted binding site for *torF* locates directly upstream of the transcriptional start site (TSS), which would exclude a binding of UdsC to *torF* mRNA. References [12,23,25,35,38,46,47,48] are cited in Supplementary Materials.

## Abbreviations

UTR	Untranslated region
EVC	Empty vector control
PCA	Principal Component Analysis
COG	Cluster of orthologous groups
NA	Not available
EMSA	Electrophoretic mobility shift assay
TBE	Tris, borate and EDTA
