# Supplementary material for: A Small RNA, UdsC, Interacts with the RpoHII mRNA and Affects the Motility and Stress Resistance of Rhodobacter sphaeroides
Source: Int J Mol Sci. 2022 Dec 7;23(24):15486. doi: 10.3390/ijms232415486 (PMC9779115; doi:10.3390/ijms232415486)
Supplement: Supplementary file 1 [file ijms-23-15486-s001.zip › ijms-2023592-supplementary.pdf]

Table S1. Bacterial strains and plasmids used in this study

Strain or plasmid	Description	Reference or source
***E. coli* strains**		
JM109	Cloning strain	New England Biolabs
S17-1	Donor strain for diparental conjugation	[1]
***R. sphaeroides* strains**		
2.4.1	wild type	[2]
2.4.1 ΔUdsC	*R. sphaeroides* 2.4.1 with a scarless deletion of *UdsC* (3′ UTR of RSP_7527)	This study
2.4.1 Δ*rpoHII*	*R. sphaeroides* 2.4.1 with a scarless deletion of *rpoHII*	[3]
2.4.1 OE UdsC	*R. sphaeroides* 2.4.1 with the plasmid PCV2_*udsC* for an inducible overexpression of sRNA UdsC	
**Plasmids**		
pJET1.2/blunt cloning vector	Ap^r, 2.97 kb	Fermentas
pBBR1	Km^r, broad-host-range cloning vector	[4]
pCV2	Gen^r, inducible overexpression plasmid	This study
pCV2_*udsC*	Gen^r, inducible overexpression of sRNA UdsC	This study
pPHU231:mVenus	Gen^r, EVC	[5]
pP-P*rpoHII*:mVenus	Sp^r, transcriptional fusion of *rpoHII* promoter sequence to mVenus	This study
pP_P*torF*:mVenus	Sp^r, transcriptional fusion of *torF* promoter sequence to mVenus	This study

Ap^r: Ampicillin, Km^r: Kanamycin, Gen^r: Gentamycin, Sp^r: Spectinomycin

Table S2. Oligodeoxynucleotide sequences for cloning, northern blot and *in vitro* transcription

Oligodeoxynucleotide	Sequence 5′-3′	Reference or source
**Cloning**		
7527_sRNA_SacI_for	CGAGAGCTCATAGTCGGCTGAGGCAGTCA	This study
7527_sRNA_mRNA_KpnI_rev	CGAGGTACCAAGAAAAAGGGCTCCGACGC	This study
7527_sRNA_up_EcoRI_for	CGAGAATTCGTTGCCGCAGGTCAGATGC	This study
7527_sRNA_up_XbaI_rev	CGATCTAGACCTCGTGCGCTCATGC	This study
7527_sRNA_dn_XbaI_for	CGATCTAGAGGGCTCCGCCGC	This study
7527_sRNA_dn_HindIII_rev	CGAAAGCTTCCGGTGCCGTTCTTGACC	This study

Bseq_torF_long_HindIII_for	ACTAAAGCTTCCTTATCGACAGGCGCGC	This study
Bseq_torF_nat_Xba_rev	ACTATCTAGAGATGATCTGGATGGCGGCCA	This study
rpoH2_Pro_HindIII_for	ACTAAAGCTTGCCCACCATCACGGTTGC	This study
rpoH2_XbaI_rev	ACTATCTAGAGGTATATCCGTCCAGTGCCAT	This study
**Northern Blot**		
UdsC	CTGCGACAATGACTGCCTCAGCCGA	[6]
5S rRNA	CTTGAGACGCAGTACCATTG	[7]
**EMSA**		
T7_RpoHII_for	TAATACGACTCACTATAGGGAGACCGGACATGTGTTTTTTCCG	This study
RpoHII_rev	AGTGCCATCAGGACCCCCTG	This study
T7_UdsC_for	TAATACGACTCACTATAGGGAGAGATAGTCGGCTGAGGCAGTC	This study
UdsC_rev	CAAAGAAAAAGGGCTCC	This study

Table S3: Log_2fold changes (calculated by DEseq2 analysis) in read counts determined by RNAseq within a UdsC overexpression strain (OE UdsC) after 15 min of IPTG treatment in comparison to the UdsC overexpression without (0 min) IPTG treatment and the UdsC knockout strain (ΔUdsC) in comparison to the wild type (WT). The Table shows the top 50 downregulated genes for the UdsC overexpression strain with IPTG (15 min) versus without IPTG (0 min).

Locus	Gene	Function	Log_2FC 15 min IPTG vs. 0 min IPTG	Log_2FC ΔUdsC vs. WT
RSP_0262	*bchX*	Chlorophyllide reductase, BchX subunit	-2.77	0,63
RSP_3706	*RSP_3706*	Hypothetical protein	-2.78	0,43
RSP_0286	*bchB*	light-independent protochlorophyllide reductase	-2.78	0,73
RSP_0104	*nuoF1*	Respiratory-chain NADH dehydrogenase,	-2.79	0,54
RSP_0102	*nuoCD*	NADH dehydrogenase (ubiquinone),	-2.79	0,72
RSP_1816	*RSP_1816*	Mg chelatase-related protein	-2.80	0,76
RSP_7559	*RSP_7559*	NA	-2.83	-0,86
RSP_2395	*RSP_2395*	cytochrome c peroxidase	-2.84	1,32
RSP_3269	*gapB*	glyceraldehyde-3-phosphate dehydrogenase	-2.85	-0,03
RSP_1284	*prkA*	Phosphoribulokinase	-2.89	-0,5
RSP_4203	*RSP_4203*	Putativ glutaredoxin family protein	-2.92	-0,59
RSP_2337	*RSP_2337*	Hypothetical protein	-2.92	-0,34
RSP_2087	*RSP_2087*	Hypothetical protein	-2.93	-0,83
RSP_0260	*bchZ*	putative chlorophyllide reductase, BchZ subunit	-2.94	0,47
RSP_0317	*hemN*	coproporphyrinogen III oxidase	-2.96	0,26
RSP_2888	*RSP_2888*	Transcriptional regulator	-2.96	0,36
RSP_7252	*RSP_7252*	Hypothetical protein	-2.96	0,25
RSP_1256	*RSP_1256*	enoyl-(acyl carrier protein) reductase	-3.02	-0,82
RSP_0278	*RSP_0278*	putative light-harvesting complex assembly protein	-3.03	0,74
RSP_0315	*pucC*	Light-harvesting 1 (B870) complex assembly	-3,12	0,74
RSP_1819	*feoA1*	Ferrous iron transport protein A	-3,12	-0,68
RSP_0285	*bchN*	Light-independent protochlorophyllide reductase	-3,13	1,09

RSP_3266	*fbpB*	Fructose-1,6-bisphosphatase	-3,14	-1,16
RSP_0284	*bchF*	2-vinyl bacteriochlorophyllide hydratase	-3,15	0,03
RSP_0261	*bchY*	Chlorophyllide reductase, BchY subunit	-3,17	1,07
RSP_0264	*crtF*	Hydroxyneurosporene methyltransferase	-3,19	1,03
RSP_1255	*RSP_1255*	Phosphate acetyltransferase	-3,19	-0,49
RSP_0279	*bchG*	Bacteriochlorophyll a synthase	-3,20	0,66
RSP_0820	*RSP_0820*	Putative cytochrome B561	-3,21	0,51
RSP_0259	*pufQ*	Protein pufQ	-3,24	0,29
NA	*3´UTR*	NA	-3,27	0,4
RSP_2758	*RSP_2758*	Hypothetical protein	-3,37	0,46
RSP_2338	*RSP_2338*	Hypothetical protein	-3,39	-0,18
RSP_0465	*RSP_0465*	Putative protease	-3,43	0,12
RSP_3044	*dorS*	DMSO/TMAO-sensor hybrid histidine kinase	-3,45	0,43
RSP_1818	*feoB*	Fe^{2+} transport system protein B	-3,46	0,43
RSP_0281	*bchE*	Mg-protoporphyrin IX monomethylester cyclase	-3,47	0,07
RSP_1817	*RSP_1817*	Hypothetical protein	-3,48	0,85
RSP_0101	*nuoB1*	NADH dehydrogenase (ubiquinone)	-3,57	0,04
RSP_2331	*ppaZ*	PpaZ, a novel pseudoazurin precursor	-3,57	-0,25
RSP_2573	*RSP_2573*	Hypothetical protein	-3,59	0,67
RSP_0464	*RSP_0464*	Putative protease	-3,62	0,23
RSP_1283	*cfxA*	Fructose-bisphosphate aldolase I	-3,65	0,16
RSP_0265	*crtE*	Geranylgeranyl pyrophosphate synthetase	-3,68	1,17
RSP_1254	*RSP_1254*	Putative acetate kinase (acetokinase)	-3,71	-0,55
RSP_7547	*RSP_7547*	Hypothetical protein	-3,82	-0,02
RSP_3788	*RSP_3788*	Hypothetical protein	-3,83	-0,32
RSP_1285	*cbbF1*	Fructose-1,6-bisphosphatase I	-4,08	-0,51
RSP_2066	*RSP_2066*	Hypothetical protein	-4,09	1,4
RSP_3434	*RSP_3434*	Hypothetical protein	-4,28	0,45
RSP_0468	*RSP_0468*	Putative 3-octaprenyl-4-hydroxybenzoate lyase	-4,47	0,37

Table S4: Log_2fold changes (calculated by DEseq2 analysis) in read counts determined by RNAseq within a UdsC overexpression strain (OE UdsC) after 15 min of IPTG treatment in comparison to the UdsC overexpression without (0 min) IPTG treatment and the UdsC knockout strain (ΔUdsC) in comparison to the wild type (WT). The Table shows the 22 and 51 genes from Figure 2D, wich are colored in red.

**Locus**	**Gene**	**Function**	**Log_2FC** 15 min IPTG vs. 0 min IPTG	**Log_2FC** ΔUdsC vs. WT
RSP_0265	*crtE*	Geranylgeranyl pyrophosphate synthetase	-3,68	1,17
RSP_0264	*crtF*	Hydroxyneurosporene methyltransferase	-3,19	1,03
RSP_0261	*bchY*	Chlorophyllide reductase, BchY subunit	-3,17	1,07
RSP_0285	*bchN*	light-independent protochlorophyllide reductase	-3,13	1,09
RSP_0315	*pucC*	Light-harvesting 1 (B870) complex assembly	-3,12	1,11
RSP_2395	*RSP_2395*	BCCP, cytochrome c peroxidase	-2,84	1,32
RSP_6125	*RSP_6125*	hypothetical protein	-2,74	1,44
RSP_6138	*RSP_6138*	hypothetical protein	-2,68	1,67
RSP_1406	*RSP_1406*	hypothetical protein	-2,49	1,21
RSP_3070	*RSP_3070*	hypothetical protein	-1,89	1,05
RSP_1480	*oppB*	ABC oligopeptide transporter	-1,87	1,74
RSP_0254	*dxsA*	1-deoxy-D-xylulose-5-phosphate synthase	-1,74	1,09
RSP_1574	*RSP_1574*	Cytochrome b562	-1,72	1,17

RSP_2085	*RSP_2085*	hypothetical protein	-1,65	1,12
RSP_6137	*RSP_6137*	transcriptional regulator, TetR family	-1,59	1,27
RSP_0293	*RSP_0293*	hypothetical protein	-1,25	1,19
RSP_0294	*RSP_0294*	hypothetical protein	-1,23	1,17
RSP_2864	*cbbE*	Pentose-5-phosphate-3-epimerase	-1,21	1,17
RSP_1278	*cbbZ*	phosphoglycolate phosphatase	-1,15	1,11
RSP_0095	*smoS*	sorbitol dehydrogenase	-1,05	1,44
RSP_0576	*RSP_0576*	Na+/solute-symporter	-1,04	1,11
RSP_0660	*RSP_0660*	hypothetical protein	-1,04	1,07
RSP_1718	*rplW*	50S ribosomal protein L23	1,09	-1,20
RSP_1733	*rpmD*	Ribosomal protein L30	1,13	-1,39
RSP_0047	*cheB2*	chemotaxis methylesterase, CheB2	1,15	-2,25
RSP_0037	*flgM*	Putative FlgM, negative regulator of flagellin	1,29	-3,16
NA	*sRNA_00061*	NA	1,41	-2,73
RSP_1049	*pheAa*	chorismate mutase	1,59	-1,26
RSP_3388	*RSP_3388*	putative Isocitrate/isopropylmalate dehydrogenase	1,60	-1,86
RSP_0042	*cheA3*	Chemotaxis histidine protein kinase, CheA3	1,67	-1,93
RSP_0044	*tlpT*	putative cytoplasmic chemoreceptor, TlpT	1,85	-2,87
RSP_0043	*cheY6*	chemotaxis response regulator, CheY6	1,99	-2,16
RSP_3386	*RSP_3386*	TRAP-T family transporter	2,12	-1,01
RSP_3389	*RSP_3389*	Isocitrate/isopropylmalate dehydrogenase	2,13	-2,40
RSP_0045	*slp*	chromosome partitioning protein	2,26	-2,31
RSP_6169	*RSP_6169*	hypothetical protein	2,48	-1,69
RSP_0046	*cheW4*	Chemotaxis protein, CheW4	2,61	-2,65
RSP_3387	*RSP_3387*	TRAP-T family transporter	2,74	-1,81
RSP_0084	*RSP_0084*	hypothetical protein	2,86	-1,67
RSP_0032	*fliA*	sigma factor FliA (Sigma-28 group, flagellar)	2,88	-1,82
NA	*UdsC*	NA	2,91	-3,46
RSP_0079	*flgF*	flagellar proximal rod protein FlgF	2,95	-2,29
RSP_0051	*torF*	Torf protein	3,02	-1,25
RSP_0073	*flgL*	flagellar hook-associated protein 3 FlgL	3,08	-2,19
RSP_0059	*fliL*	flagellar biosynthesis protein, FliL	3,15	-2,40
RSP_0076	*flgI*	flagellar P-ring protein	3,15	-2,57
RSP_0054	*fliG*	Probable flagellar motor switch protein FliG	3,25	-1,39
RSP_0065	*fliR*	Flagellar protein FliR	3,29	-1,65
RSP_6086	*RSP_6086*	hypothetical protein	3,33	-2,04
RSP_6092	*RSP_6092*	hypothetical protein	3,34	-2,29
RSP_0081	*flgD*	Flagellar scaffolding protein FlgD	3,35	-2,82
RSP_0064	*fliQ*	Flagellar protein FliQ	3,38	-2,71
RSP_0074	*flgK1*	FlgK flagellar hook-associated protein 1	3,40	-2,44
RSP_0033	*RSP_0033*	hypothetical protein	3,40	-2,59
RSP_0080	*flgE*	flagellar hook protein FlgE	3,41	-2,98
RSP_0077	*flgH*	Flagellar L-ring protein	3,48	-2,08
RSP_0072	*RSP_0072*	possible invasion protein	3,50	-2,42
RSP_0078	*flgG*	Flagellar distal rod protein	3,51	-2,74
RSP_0231	*motB*	flagellar MotB protein	3,51	-2,70
RSP_6093	*flgJ*	FlgJ	3,53	-1,83
RSP_0060	*fliM*	Flagellar switch protein FliM	3,54	-2,20
RSP_0066	*flhB*	Flagellar protein FlhB	3,60	-2,32
RSP_0062	*fliO*	Flagellar protein FliO	3,74	-2,64
RSP_0058	*fliK*	FliK, flagellar hook-length control protein	3,77	-2,01

RSP_0233	*motA*	Flagellar motor protein MotA	3,77	-1,40
RSP_0082	*flgC*	flagellar basal-body rod protein FlgC	3,83	-2,77
RSP_0035	*RSP_0035*	hypothetical protein	3,87	-2,56
RSP_0067	*RSP_0067*	hypothetical protein	3,89	-2,80
RSP_0061	*fliN*	Flagellar motor switch FliN protein	3,97	-2,08
RSP_0063	*fliP*	Flagellar transport protein FliP	4,00	-2,08
RSP_0036	*flgA*	flagellar basal-body P-ring formation protein FlgA	4,02	-2,49
RSP_0034	*flhA*	flagellar biosynthesis protein, FlhA	4,05	-2,48
RSP_0083	*flgB*	Flagellar proximal rod protein FlgB	4,65	-2,19

Table S5: Log_2fold changes (calculated by DEseq2 analysis) in read counts determined by RNAseq within a UdsC overexpression strain (OE UdsC) after 15 min of IPTG treatment in comparison to the UdsC overexpression without (0 min) IPTG treatment and the UdsC knockout (ΔUdsC) in comparison to the wild type (WT). The Table shows 27 genes of the motility gene cluster that are not or slightly regulated.

**Locus**	**Gene**	**Function**	**Log_2FC** 15 min IPTG vs. 0 min IPTG	**Log_2FC** ΔUdsC vs. WT
RSP_1303	*flgE1*	putative flagellar hook protein	-0,13	-0,34
RSP_1304	*RSP_1304*	flagellar hook-associated protein	-1,33	-0,31
RSP_1305	*RSP_1305*	putative flagellar hook-associated protein	-0,28	-1,04
RSP_1307	*flgI1*	flagellar P-ring protein precursor	-0,35	-0,94
RSP_1306	*RSP_1306*	PAS sensor Signal Tranduction Histidine Kinase	0,76	0,03
RSP_1308	*RSP_1308*	hypothetical protein	-0,71	0,18
RSP_1309	*fliP1*	Type III secretion system inner membrane P protein	0,03	-0,91
RSP_1310	*RSP_1310*	hypothetical protein	0,59	1,07
RSP_1311	*fliH1*	putative flagellar biosynthesis/type III secretory pathway protein	-0,97	1,80
RSP_1312	*fliF*	putative flagellar M-ring protein	0,24	-0,39
RSP_1313	*RSP_1313*	possible flagellar synthesis protein	-0,67	-0,44
RSP_1314	*RSP_1314*	hypothetical protein	-0,85	-0,08
RSP_1315	*RSP_1315*	hypothetical protein	-1,31	0,60
RSP_1316	*motA1*	flagellar motor protein	0,69	-0,25
RSP_1318	*RSP_1318*	hypothetical protein	0,04	-0,18
RSP_1319	*RSP_1319*	possible invasion protein	0,06	-0,19
RSP_1320	*flhA1*	Flagellar biosynthesis pathway, component FlhA	-0,02	-0,80
RSP_1321	*fliR1*	Flagellar biosynthesis pathway, component FliR	-0,35	-1,46
RSP_1322	*fhlB*	flagellar biosynthesis pathway, component FlhB	-0,17	-0,21
RSP_6155	*RSP_6155*	hypothetical protein	0,36	0,53
RSP_1323	*RSP_1323*	hypothetical protein	0,23	0,84
RSP_1324	*flgH1*	flagellar L-ring protein precursor	0,32	0,48
RSP_1325	*RSP_1325*	putative flagellar basal-body P-ring formation protein FlgA	-0,40	0,30
RSP_1326	*RSP_1326*	putative flagellar basal body rod protein	-0,41	0,22
RSP_1327	*RSP_1327*	putative flagellar basal body rod protein	0,07	0,04
RSP_1328	*RSP_1328*	flagellar biosynthetic protein FliQ	0,16	0,25
RSP_1329	*fliE1*	putative flagellar hook-basal body complex protein	-0,81	-1,31

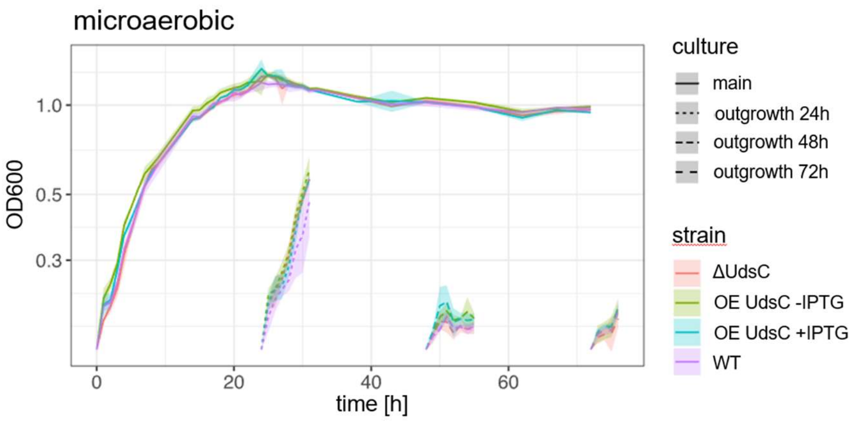

Figure S1: Growth curves of WT, UdsC knockout strain (ΔUdsC) and the UdsC overexpression strain (OE UdsC strain (with or without IPTG added) under microaerobic conditions. The cultures were incubated for 72 h. 24, 48 and 72 h after inoculation, part of the cultures was diluted into fresh medium and outgrowth was monitored, while the remaining culture was further incubated for monitoring the OD. The lines represent the mean of biological triplicates and the standard deviations are indicated (shade).

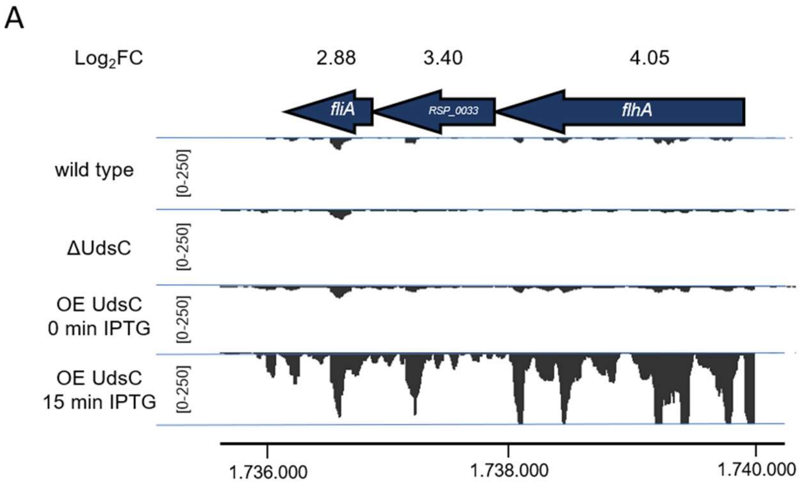

B

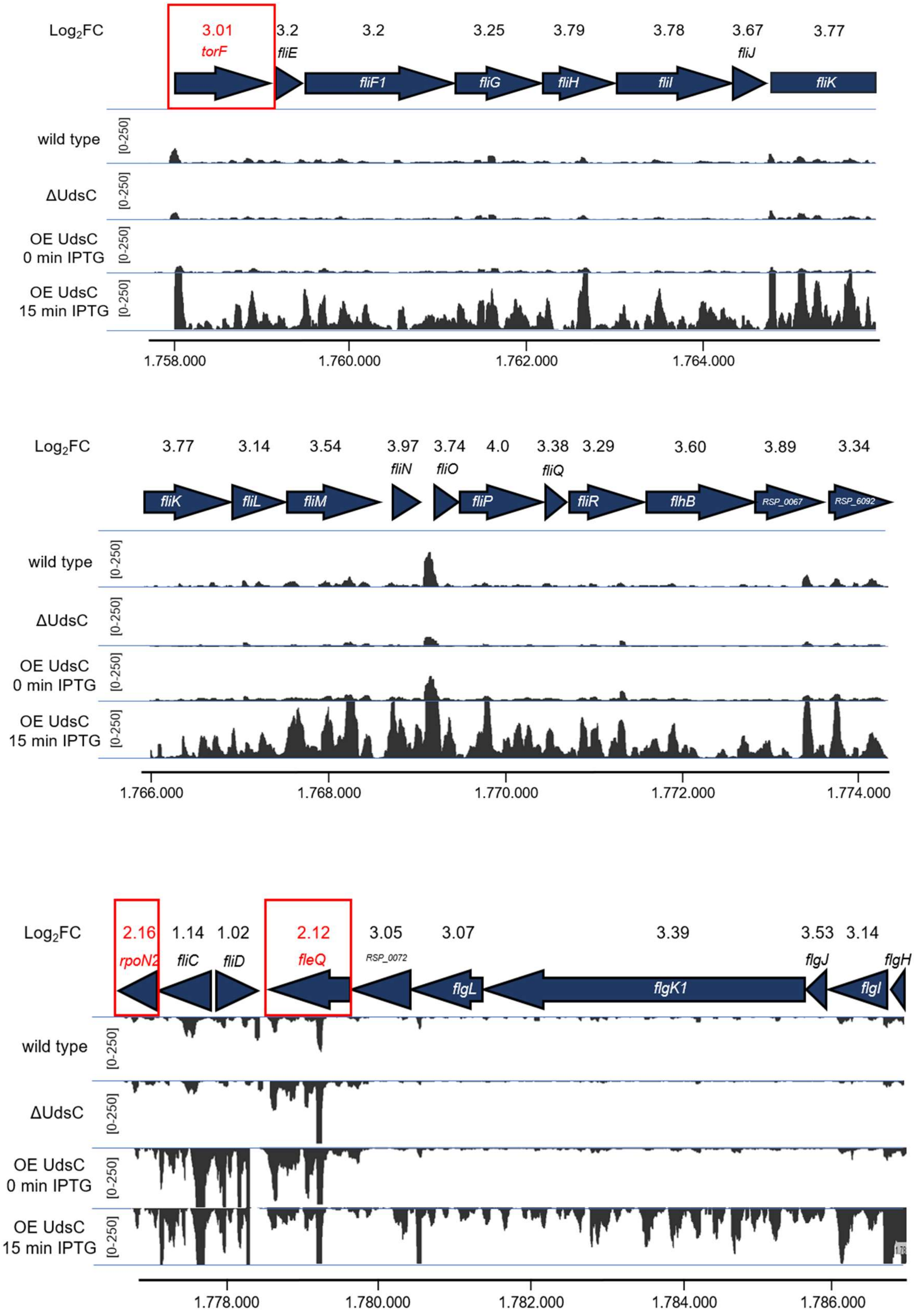

Log2FC
3.01
torF
3.2
fliE
3.2
fliF1
3.25
fliG
3.79
fliH
3.78
fliI
3.67
fliJ
3.77
fliK
wild type
ΔUdsC
OE UdsC 0 min IPTG
OE UdsC 15 min IPTG
[0-250]
1.758.000
1.760.000
1.762.000
1.764.000
Log2FC
3.77
fliK
3.14
fliL
3.54
fliM
3.97
fliN
3.74
fliO
4.0
fliP
3.38
fliQ
3.29
fliR
3.60
flhB
3.89
RSP_0067
3.34
RSP_6092
wild type
ΔUdsC
OE UdsC 0 min IPTG
OE UdsC 15 min IPTG
[0-250]
1.766.000
1.768.000
1.770.000
1.772.000
1.774.000
Log2FC
2.16
rpoN2
1.14
fliC
1.02
fliD
2.12
fleQ
3.05
RSP_0072
3.07
flgL
3.39
flgK1
3.53
flgJ
3.14
flgI
flgH
wild type
ΔUdsC
OE UdsC 0 min IPTG
OE UdsC 15 min IPTG
[0-250]
1.778.000
1.780.000
1.782.000
1.784.000
1.786.000

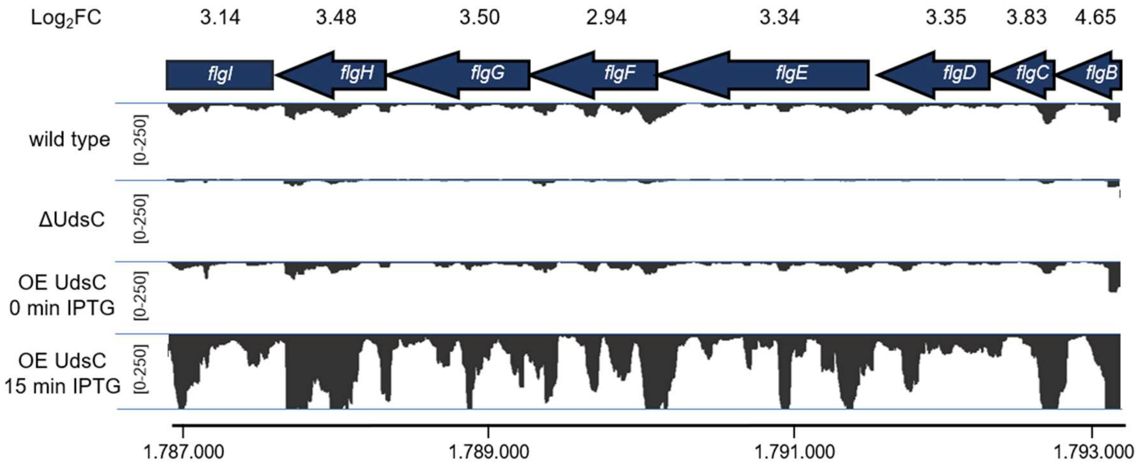
Log2FC
3.14
3.48
3.50
2.94
3.34
3.35
3.83
4.65
flgI
flgH
flgG
flgF
flgE
flgD
flgC
flgB
wild type
[0-250]
ΔUdsC
[0-250]
OE UdsC 0 min IPTG
[0-250]
OE UdsC 15 min IPTG
[0-250]
1.787.000
1.789.000
1.791.000
1.793.000

C

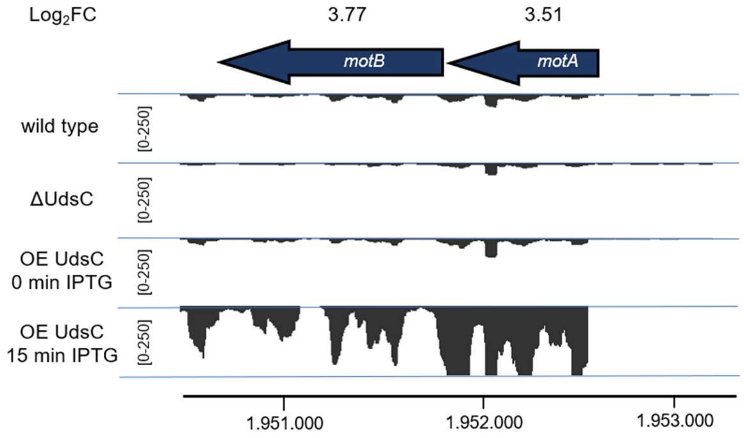
Log2FC
3.77
3.51
motB
motA
wild type
[0-250]
ΔUdsC
[0-250]
OE UdsC 0 min IPTG
[0-250]
OE UdsC 15 min IPTG
[0-250]
1.951.000
1.952.000
1.953.000

D

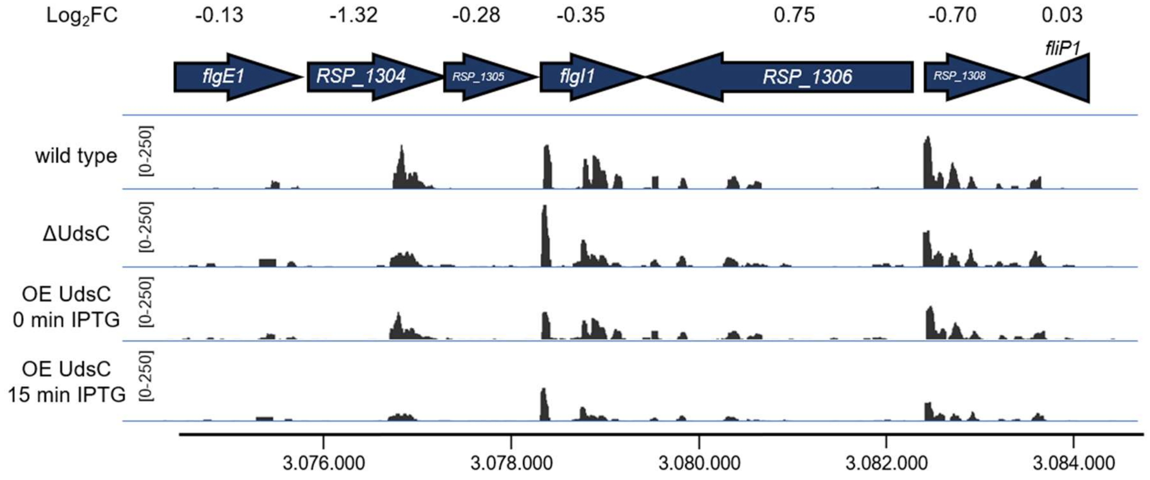
Log2FC
-0.13
-1.32
-0.28
-0.35
0.75
-0.70
0.03
fliP1
flgE1
RSP_1304
RSP_1305
flgI1
RSP_1306
RSP_1308
wild type
[0-250]
ΔUdsC
[0-250]
OE UdsC 0 min IPTG
[0-250]
OE UdsC 15 min IPTG
[0-250]
3.076.000
3.078.000
3.080.000
3.082.000
3.084.000

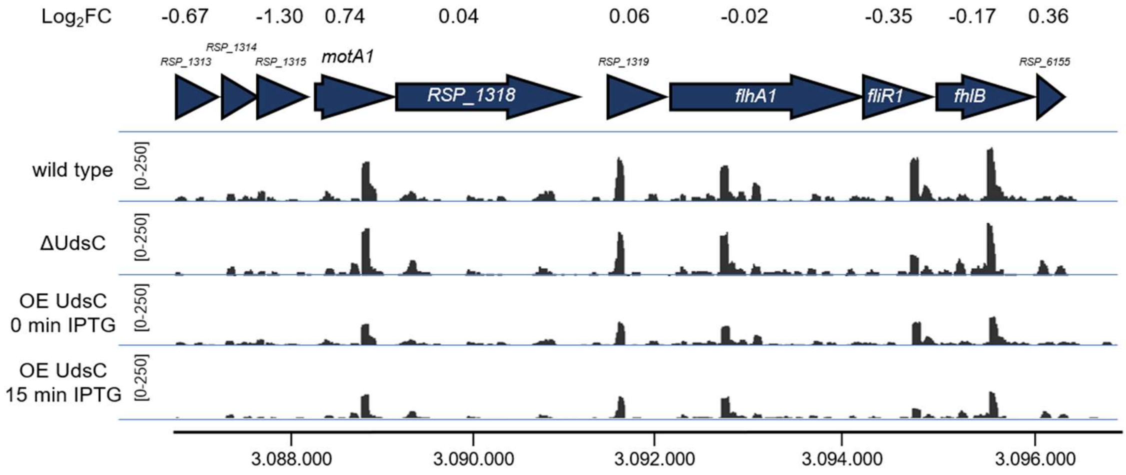

Figure S2: Effect of UdsC on expression of the motility genes from RNAseq visualized by the Integrated Genome Browser. Wild type, UdsC deletion strain (ΔUdsC) and UdsC overexpression (OE UdsC) with and without IPTG (0 min IPTG or 15 min IPTG) were cultivated under microaerobic conditions and total RNA was isolated for RNA sequencing as described in materials and methods. The Log_2fold change (Log_2FC) of UdsC overexpression strain (OE UdsC) with IPTG (15 min) versus without IPTG (0 min) is idicated. Genes for important regulators of the flagella genes are marked in red.

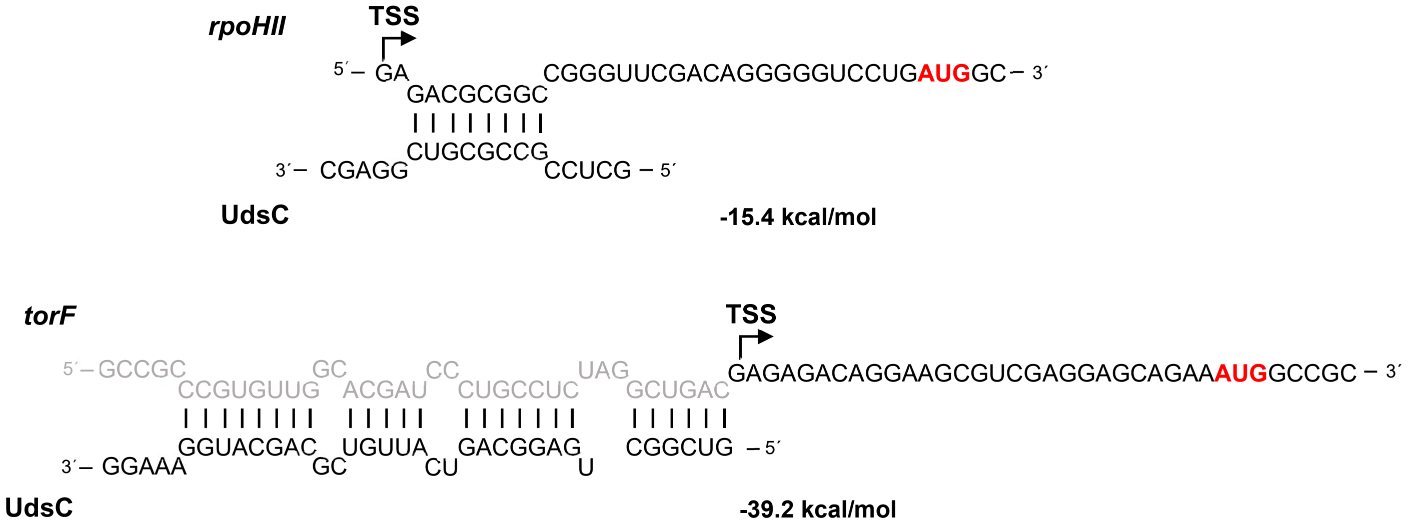

Figure S3: (**A**) Predicted base pairing between UdsC and *rpoHII* mRNA transcript by IntaRNA webtool [8]. The transcription start site (TSS) is indicated by an arrow and the start codon AUG is shown in red. (**B**) The predicted binding site for *torF* locates directly upstream of the transcriptional start site (TSS), which would exclude a binding of UdsC to *torF* mRNA.

[1] Simon, R., O´Connell, M., Labes, M., and Puhler, A. (1986) Plasmid vectors for the genetic analysis and manipulation of rhizobia and other gram-negative bacteria, *Methods in enzymology* **118**, 640-659.

[2] Van Niel, C.B. (1944) General Physiology, Morphology, and Classification of the Non-Sulfur Purple and Brown Bacteria, *Bacteriological Reviews*, **8**, 1-118

[3] Nuss, A.M., Glaeser, J., and Klug, G. (2009) RpoH(II) activates oxidative-stress defense systems and is controlled by RpoE in the singlet oxygen-dependent response in *Rhodobacter sphaeroides*. *J. Bacteriol*., **191**, 220-230.

[4] Kovach, M.E.; Elzer, P.H.; Hill, D.S.; Robertson, G.T.; Farris, M.A.; Roop, R.M.; Peterson, K.M. Four new derivatives of the broad-host-range cloning vector pBBR1MCS, carrying different antibiotic-resistance cassettes. *Gene* **1995**, *166*, 175–176, doi:10.1016/0378-1119(95)00584-1.

[5] Grützner, J., Remes, B., Eisenhardt, K. M. H., Scheller, D., Kretz, J., Madhugiri, R., et al. (2021). sRNA-mediated RNA processing regulates bacterial cell division. *Nucleic Acids Res* 49, 7035–7052. doi: 10.1093/nar/gkab491

[6] Spanka, D.-T., and Klug, G. (2021). Maturation of UTR-Derived sRNAs Is Modulated during Adaptation to Different Growth Conditions. *Int J Mol Sci* 22. doi: 10.3390/ijms222212260

[7] Berghoff, B.A., Glaeser, J., Sharma, C.M, Zobawa, M., Lottspeich, F., Vogel, J., and Klug, G. (2011) Contribution of Hfq to photooxidative stress resistance and global regulation in *Rhodobacter sphaeroides*. *Mol. Microbiol.*, **80**, 1479-1495.

[8] Mann, M.; Wright, P.R.; Backofen, R. IntaRNA 2.0: enhanced and customizable prediction of RNA-RNA interactions. *Nucleic Acids Res.* **2017**, *45*, W435-W439, doi:10.1093/nar/gkx279.
